# Erratum for Gebo et al., “Early antibody treatment, inflammation, and risk of post-COVID conditions”

**DOI:** 10.1128/mbio.02979-23

**Published:** 2023-12-14

**Authors:** Kelly A. Gebo, Sonya L. Heath, Yuriko Fukuta, Xianming Zhu, Sheriza Baksh, Allison G. Abraham, Feben Habtehyimer, David Shade, Jessica Ruff, Malathi Ram, Oliver Laeyendecker, Reinaldo E. Fernandez, Eshan U. Patel, Owen R. Baker, Shmuel Shoham, Edward R. Cachay, Judith S. Currier, Jonathan M. Gerber, Barry Meisenberg, Donald N. Forthal, Laura L. Hammitt, Moises A. Huaman, Adam Levine, Giselle S. Mosnaim, Bela Patel, James H. Paxton, Jay S. Raval, Catherine G. Sutcliffe, Shweta Anjan, Thomas Gniadek, Seble Kassaye, Janis E. Blair, Karen Lane, Nichol A. McBee, Amy L. Gawad, Piyali Das, Sabra L. Klein, Andrew Pekosz, Evan M. Bloch, Daniel Hanley, Arturo Casadevall, Aaron A. R. Tobian, David J. Sullivan, on behalf of the CSSC-004 Consortium

## ERRATUM

Volume 14, no. 5, e00618-23, 2023, https://journals.asm.org/doi/10.1128/mbio.00618-23. Page 6, Table 1, last row: “3,041 (2,197, 4,208)” should read “13,774 (8,665, 21,896),” and “13,774 (8,665, 21,896)” should read “3,041 (2,197, 4,208).”

